# Jet‐Printing Microfluidic Devices on Demand

**DOI:** 10.1002/advs.202001854

**Published:** 2020-10-26

**Authors:** Cristian Soitu, Nicholas Stovall‐Kurtz, Cyril Deroy, Alfonso A. Castrejón‐Pita, Peter R. Cook, Edmond J. Walsh

**Affiliations:** ^1^ Osney Thermofluids Institute Department of Engineering Science University of Oxford Osney Mead Oxford OX2 0ES UK; ^2^ Department of Engineering Science University of Oxford Parks Road Oxford OX1 3PJ UK; ^3^ The Sir William Dunn School of Pathology University of Oxford South Parks Road Oxford OX1 3RE UK; ^4^ Iota Sciences Ltd. Begbroke Science Park, Begbroke Oxfordshire OX5 1PF UK

**Keywords:** cell cloning, fluid walls, immiscible jet, microfluidics, Poisson distribution

## Abstract

There is an unmet demand for microfluidics in biomedicine. This paper describes contactless fabrication of microfluidic circuits on standard Petri dishes using just a dispensing needle, syringe pump, three‐way traverse, cell‐culture media, and an immiscible fluorocarbon (FC40). A submerged microjet of FC40 is projected through FC40 and media onto the bottom of a dish, where it washes media away to leave liquid fluorocarbon walls pinned to the substrate by interfacial forces. Such fluid walls can be built into almost any imaginable 2D circuit in minutes, which is exploited to clone cells in a way that beats the Poisson limit, subculture adherent cells, and feed arrays of cells continuously for a week. This general method should have wide application in biomedicine.

## Graphical abstract

There is an unmet demand for microfluidics in biomedicine. This paper describes a contactless method to fabricate microfluidic devices on demand on standard Petri dishes using microjets. Circuits with almost any imaginable 2D shape are built in minutes, which is exploited to beat the Poisson limit in cloning applications, subculture adherent cells, and feed arrays of cells continuously for a week.

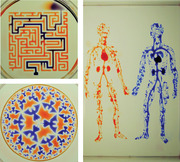

## 1 Introduction

As sensitivities of methods for detecting biomolecules improve, demand for handling ever‐smaller volumes increases—and this drives development of microfluidic approaches.^[^ ^1^, ^2^, ^3^ ^]^ However, few of these are found in biomedical workflows, with the exception of those involving microplates. Why? Reasons given include: devices are expensive and take days/weeks to make, they are complicated to operate, their contents are inaccessible, and they are not made with cell‐friendly materials.^[^ ^1^ ^]^

In the everyday world, gravity is such a dominant force that most objects are made with solids, and one cannot contemplate building them out of liquids, which just collapse into puddles. Consequently, liquids are always contained by solid walls, otherwise they drain away. In the microworld, gravity becomes irrelevant, and interfacial forces dominate (think of water striders skimming over ponds, and dewdrops sticking to blades of grass). Consequently, an interface between two fluids can act as a robust wall separating the two. This paradigm enabled the emergence of open microfluidics,^[^ ^4^ ^]^ where solid walls are replaced by air:water^[^ ^5^ ^]^ or oil:water interfaces.^[^ ^6^, ^7^, ^8^ ^]^

Open microfluidic devices are generally easier to integrate into biomedical workflows as they offer better optical and physical access to samples, reduced adhesion of reagents to solid surfaces and are resistance to blocking by air bubbles. However, despite the ever‐increasing efforts to simplify manufacturing workflows for such devices, most of them still require etching of the substrate,^[^ ^9^ ^]^ surface treatment^[^ ^10^ ^]^ or contact,^[^ ^7^ ^]^ or some combination of these^[^ ^8^ ^]^ to confine fluidic structures. Such complex manufacturing processes deter many biologists who favor fast flexible prototyping without having to compromise biocompatibility.^[^ ^11^ ^]^ Recently, microfluidic arrangements were created simply by dragging a Teflon rod/stylus resting on the surface of a dish to reshape cell‐culture media and an immiscible overlay into the wanted pattern.^[^ ^7^ ^]^ However, motion of the stylus relative to its holder introduced play in the *x*–*y* plane, reducing accuracy and precision. Moreover, proteins in media aggregate on the stylus to reduce reproducibility and increase risks of cross contamination. Additionally, the stylus needs frequent change due to wear. All these drawbacks stem from the effects of contact.

Here, we describe a contactless method to fabricate microfluidic devices on demand, where the only “building” materials used are those in the biocompatible trio—cell media, the immiscible fluorocarbon FC40, and a polystyrene Petri dish. FC40 is “jetted” from a dispensing needle through bulk FC40 and media on to the untreated bottom of a dish. Complex microfluidic structures with features <50 µm in size can be produced reproducibly with high accuracy in minutes. The aqueous phase is confined by fluid walls—media:FC40 interfaces—which are robust yet easily pierced (so liquids can be added/removed through them at any preselected point) whilst being transparent. The physics underlying flow during such jetting is complex;^[^ ^12^, ^13^, ^14^, ^15^ ^]^ therefore, we establish appropriate conditions. We then exploit jetting to “beat” the Poisson limit to clone single mammalian cells, subculture them (again using a contactless method), and perfuse them steadily with fresh media for a week.

## 2 Results

### 2.1 Approach

**Figure 1:**
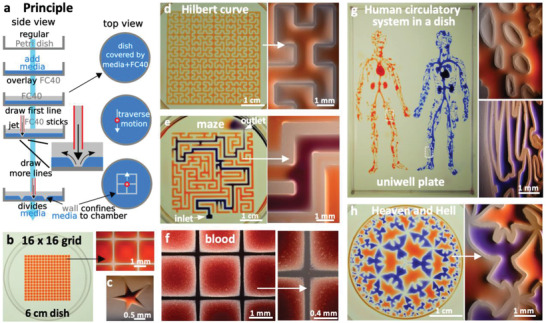
Principle. a) A “microjet” of an immiscible fluorocarbon (FC40) is projected through FC40 and media on to the bottom of a dish; media is pushed aside, and FC40 sticks to the bottom (it wets polystyrene better than media). Moving the jet now draws lines to form a grid with FC40 walls. b) A 16 × 16 grid (interchamber spacing 1.9 mm; 600 nL red dye added to each chamber; zoom shows fluid walls). c) Star‐shaped walls (red dye added after jetting). d) A square with one internal wall shaped like a Hilbert curve (a continuous line with many 90° and 45° turns). Red dye was added at many points; it diffused around the wall throughout the square. Zoom shows some turns. e) Path through a maze with a single inlet and outlet. The circuit was built by jetting FC40 through media plus red dye; when blue dye is pumped into the inlet, it takes the path of least resistance through the maze. Zoom shows some walls. f) Part of 16 × 16 grid where FC40 is jetted through blood instead of media. Zoom shows the jet leaves no cells in what are now FC40 walls. g) Human circulatory system in a uniwell plate with dimensions of a 96‐well plate. Red and blue dyes were infused into major “arteries” and “veins” which then flow/diffuse throughout each system. Zooms show regions in white rectangles. h) Walls were built in design inspired by “Circle Limit IV (Heaven and Hell)” by M.C. Escher – WikiArt. Patterns for Figure 1d,e,g were obtained from the following sources: © Wikimedia Commons (author Braindrain0000), Can Stock Photo Inc. (author Taui), pngegg.com, respectively. Images used to inspire Figure 1d and 1e were obtained and used with permission from Wikimedia Commons (name "Hilbert curve.png", author Zbigniew Fiedorowicz) and Can Stock Photo Inc. (image number csp49511651, author Taui)”. Image used to inspire Figure 1g is a public domain image. Image used to inspire Figure 1h was from Sazdanovic et al. ^[^ ^16^ ^]^ and is used under the terms of the CC‐BY license, Copyright 2012, Radmila Sazdanovic, published by MDPI.

**Figure** **1**a illustrates the approach. The bottom of a standard tissue‐culture dish is covered with a film of cell‐growth media, and an FC40 overlay added to prevent evaporation. A dispensing needle filled with FC40, connected to a syringe pump, and held by a three‐way traverse is now lowered below the surface of the fluorocarbon; starting the pump jets FC40 on to the dish to push media aside. As FC40 has a low equilibrium contact angle (CA) on polystyrene (<10°), it wets it better than media (equilibrium CA ≈50°),^[^ ^6^ ^]^ so it adheres to the bottom. Moving the microjet sideways then creates a line of FC40 on the dish, and drawing more lines creates a grid with 256 chambers in <2 min (Figure 1b; Movie S1, Supporting Information). Each chamber is isolated from others by liquid walls of FC40 pinned to polystyrene. Interfacial forces dictate chamber geometry—a spherical cap sitting on a square footprint (height ≈75 µm; volume ≈100 nL). Up to ≈900 nL more media can be pipetted into chambers as fluid walls morph above unchanging footprints. Chambers are then used like wells in microplates: liquids are added/removed to/from them by pipetting through FC40 instead of air. The maximum and minimum volumes that can be held in chambers without altering footprints are determined by advancing and receding contact angles; addition of too much media inevitably merges adjacent chambers. Even so, chambers accept a manyfold wider range of volume than equally spaced wells in a microplate, whilst containing ≈1000th the volume.^[^ ^7^ ^]^ Consequently, if chambers contain cells, the volume ratio of intra‐ to extracellular fluid more closely resembles that in vivo. Importantly, this method is contactless: the nozzle touches neither dish nor media. Moreover, one pipet tip can add/extract reagents to/from many chambers without detectable cross‐contamination (shown—for example—by seeding bacteria in every other chamber, adding media to all through one tip, and finding that bacteria grow only in inoculated chambers as others remain sterile).^[^ ^7^ ^]^ In other words, a tip is washed effectively by passage through FC40 between chambers, and—when using cells—we make doubly sure by additionally washing in 70% ethanol.

Jetting allows walls to be built with a precision only limited by that of the traverse—illustrated by building a star‐shaped chamber (Figure 1c), a square enclosing a single continuous wall shaped like a Hilbert curve (Figure 1d; fabrication time ≈5 min), and a microfluidic maze where blue dye infused into the inlet solves the problem of finding the path to the sole exit (Figure 1e; conduits have maximum widths of 1 mm, and heights of ≈350 µm). Reproducibility is demonstrated by constructing a fractal circuit in which a central input is connected through conduits (footprint width 450 µm, maximum height ≈160 µm) that each make five right‐angled turns to one of 64 outlets; dye infused into the input reaches all outlets simultaneously, showing that conduit cross‐sections and lengths are similar throughout (Figure S1, Supporting Information). The method is not restricted to media, which can be replaced by whole human blood (Figure 1f). Nor is it restricted to regular patterns, illustrated by the “human circulatory system” (Figure 1g; jetting time ≈90 min) and design inspired by “Circle Limit IV (Heaven and Hell)” by M.C. Escher, WikiArt, (Figure 1h; jetting time ≈5 min). These circuits emphasize the ability to reproduce almost any pre‐existing pattern with features of ever‐decreasing size (“veins” or “angels”). In these and subsequent examples, dyes are added solely to aid visualization; they play no role in stabilizing liquid structures. We also use “media” to describe DMEM plus 10% FBS unless stated otherwise. In addition, fluid walls are sufficiently stable to be carried up/downstairs between labs, incubators, and microscopes—just like any dish filled with liquid (see Movie S2 in the Supporting Information). These examples illustrate the versatility of jetting and show that circuits with almost any imaginable 2D shape can be built quickly and accurately using cell‐friendly materials.

### 2.2 Building Walls and Conduits with Different Widths

**Figure 2:**
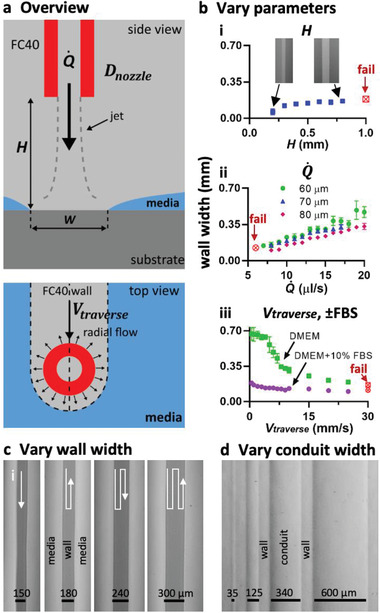
Varying jetting parameters. Unless stated otherwise throughout the paper, $\dot{Q}$ = 8 µL s^−1^, *H* (height of nozzle above substrate) = 0.4 mm, *D_i_* (diameter of jetting nozzle) = 60 µm, *V*_traverse_ (lateral traverse speed) = 10 mm s^−1^, and media was DMEM + 10% FBS. a) Overview. A submerged FC40 microjet drives media off the substrate to create an FC40 wall, and some parameters varied are indicated. b) Controlling wall width (red symbols: continuous walls fail to form) by varying: i) jet height (inset: images of walls when *H* = 0.2 and 0.8 mm). The jet width increases with distance from the nozzle, so lowering the nozzle yields progressively narrower wall. ii) Flow rate and nozzle diameter. Wall width increases as jet momentum increases (by increasing flowrate or reducing nozzle diameter). iii) Traverse velocity and serum content. Proteins in the serum causes an increased adhension force at the pinning lines, so the “sweeping” action of the jet momentum is diminished, resulting in smaller wall widths. c) Varying wall width by overlapping walls. Wall width can be increased by setting the separation between jetting paths to be smaller than the wall width generated by one pass. d) Making conduits with different widths (walls all have equal widths) by varying the distance between consecutive paths. *H* = 0.3 mm, $\dot{Q}$ = 5 µL s^−1^, *D*_nozzle_ = 60 µm, and *V*_traverse_ = 1.8 mm s^−1^.

In theory, many parameters including interfacial properties, jet momentum, densities, and kinematic viscosities influence wall stability and shape. FC40 has a density of 1.855 g mL^−1^, and intuition suggests that buoyancy should force media above the fluorocarbon; however, media remains pinned to a prewetted dish as interfacial forces are stronger than buoyancy forces. Other parameters were varied to assess their impact on wall construction (**Figure** **2**a; Figure S2a, Supporting Information). When the nozzle is high above the substrate (i.e., *H* is large), no walls form; the momentum of the jet falls as it travels through FC40 (which has a kinematic viscosity of 2.2 cSt compared to 1 for water), making it insufficient to displace media (Figure 2bi, red symbol). Lowering the nozzle decreases the loss to the point when the final jet momentum that hits the substrate is large enough to push media aside. FC40 then remains pinned to the substrate to give a wall. Examination of streamlines (Movie S3, Supporting Information) shows that jet width increases with distance from the nozzle, so further lowering yields progressively narrower walls as the jet impinges on progressively smaller substrate areas. Even so, wall width proves to be relatively tolerant to changes in *H* (Figure 2bi), so walls can be constructed using a nozzle positioned at the same height above dishes from different manufacturers that turn out to be slightly bowed to different degrees. At a low volumetric flow rate ($\dot{Q}$), momentum is insufficient and no wall forms; increasing $\dot{Q}$ yields walls with increasing width (Figure 2bii). Nozzle diameter (*D*_nozzle_), traverse velocity (*V*_traverse_) and interfacial properties (especially presence of FBS) all affect wall width (Figure 2bii,iii; see Figure S2b (Supporting Information) for data on E8+, mTesR, and StemFlex media). Wider walls can be made by overlapping jetting lines (Figure 2c). Conduit width is easily adjusted down to at least 35 µm by jetting walls closer together (Figure 2d). We have therefore identified a range of conditions allowing wall formation even on commercially available dishes that are not completely flat.

### 2.3 Controlling Flow through Circuits

Flow through these circuits can easily be driven by external pumps; fluid walls/ceilings spontaneously seal around connecting hydrophilic tubes on insertion, and then morph above unchanging footprints during flow. However, adding external pumps adds complexity and cost. As intrinsic differences in Laplace pressure can also drive flow through microfluidic circuits,^[^ ^6^, ^17^ ^]^ we demonstrated this in these circuits (Figure S3, Supporting Information).

Thus far, we have jetted FC40. We now create a microfluidic “valve” by jetting media (Figure S4 and Movie S4, Supporting Information). We first build a conduit with a dead end (using an FC40 jet), and make a hole through the end wall (using a media jet traversing down the conduit axis) to allow flow through the hole. We next rebuild the original end wall (using an FC40 jet that traverses across the conduit) to stop flow. Such valves can be opened and closed as many times as needed.

### 2.4 “Beating” the Poisson Limit during Cell Cloning

Mammalian cells are often cloned by splitting a dilute cell suspension amongst wells in a microplate so most wells get no cells, a few get one, and fewer still get two or more—before singletons are selected, grown, and resulting colonies picked. This approach is wasteful, as Poisson statistics ensure that wells with singletons are in a minority. Although many approaches (including fluorescence activated cell sorting and combinations of printing plus imaging)^[^ ^18^ ^]^ can increase the number of wells with singletons and so “beat” the Poisson limit, cost and complexity prevent widespread adoption.^[^ ^19^, ^20^ ^]^ Moreover, solid plastic walls around each well in a microplate yield “edge effects” that obscure cells close to walls.^[^ ^21^ ^]^ Such effects are so severe that many users never check to see whether a well contains just one cell immediately after plating, and go through a second cloning round to increase the chances of achieving monoclonality. This prompted development of an approach that allies jetting with use of Voronoi diagrams.

**Figure 3:**
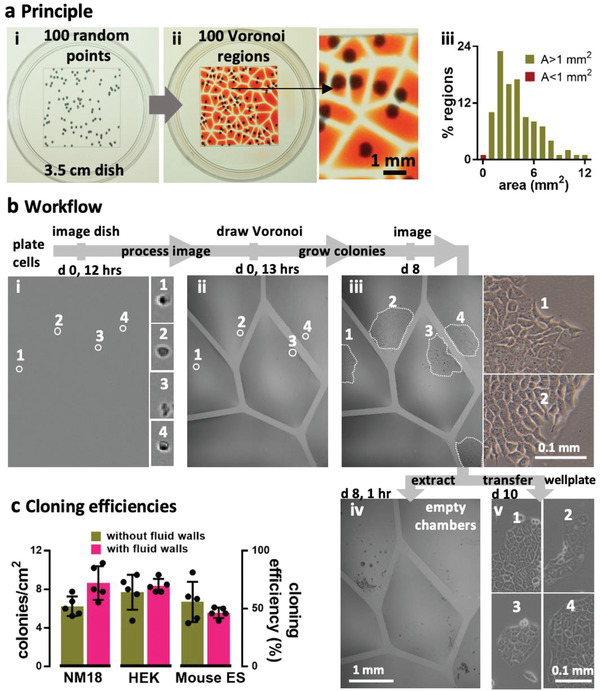
Beating the Poisson limit during cell cloning. a) Principle illustrated using 100 randomly distributed dots in a 2 cm square printed on clear film placed under a 35 mm dish. i) Dish + dots. ii) After recording dot positions, the Voronoi diagram is determined, polygonal walls built around each dot, and each polygonal chamber filled with dye (zoom shows some chambers). iii) Distribution of areas in this Voronoi diagram. Two polygons had area (*A*) <1 mm^2^, a threshold determined by the minimum area (hence volume) the infusing needle can access. b) Workflow illustrated using NM18 cells (12 cells cm^−2^ or ≈48 in 2 × 2 cm square) plated in a 35 mm dish. Phase‐contrast images are of the same region, and numbered zooms show magnifications of different founder cells and their colonies. i) Cells 1–4 12 h after plating. ii) The same cells after jetting surrounding polygons. iii) By day 8, cells develop into colonies (outlined by dotted lines). iv) Emptied polygons (achieved by adding/removing PBS, adding trypsin, incubation, and removing most cells). Overemptying some chambers leaves FC40 (dark blobs) attached to the bottom. v) After removal, each clone was reseeded in a well in a 12‐well plate, and imaged 2 days later. c) Cloning efficiencies. Each cell type was seeded (12 cells cm^−2^) in 10 × 35 mm dishes, colonies counted after 8 days, and cloning efficiencies calculated. Five dishes were used to assess cloning efficiencies conventionally (“without fluid walls”), and five using polygons (“with fluid walls”). There was no or little significant difference between the two approaches (unpaired t test for HEK, ES, and NM18 gave *p* = 0.488, 0.3, and 0.033, respectively).

The approach is illustrated using 100 randomly distributed dots as cell surrogates printed on a transparency stuck on the underside of a dish (**Figure** **3**ai). After imaging and mapping dot positions, we build an analogue of a “cloning ring” around each dot by jetting surrounding FC40 walls. Instead of jetting circles, we compute the relevant Voronoi diagram—a set of polygons where each contains one dot—and jet polygonal walls around dots (Figure 3aii; Figure S5a, Supporting Information). More fluid can be added to/retrieved from such polygons, within the bounds of advancing and receding contact angles (Movie S5, Supporting Information). Then, the Poisson limit is “beaten” in the sense that every polygon contains one dot. In practice, some polygons prove unusable—FC40 walls have a thickness (*W*) and two dots may lie <*W* apart (which is too small to accommodate a new wall), and nozzle width may be greater than polygon width (so polygons merge when media is added subsequently). The fraction of such lost polygons inevitably increases as dot density increases; fortunately, densities used for cloning ensure few are lost (e.g., only 2% with 100 dots/cells per 2 cm square; Figure 3aiii). Importantly, the user has the freedom to set any lower and/or upper limits on the acceptable areas. An alternative approach also involves jetting a circular or square wall around each cell to give chambers with similar volumes, but this yields many fewer isolated clones per dish than the numbers achieved here.

Figure 3b illustrates an experiment. Mouse NM18 cells are plated, imaged (Figure 3bi), positions of viable cells identified, the Voronoi diagram computed, and polygons jetted around each living cell (Figure 3bii). Once colonies grow (Figure 3biii), trypsin is added to polygons, cells removed (Figure 3biv) and transferred to standard 12‐well plates, and clones expanded (Figure 3biv). Cloning efficiencies with three mammalian cells (NM18, mouse ES, human embryonic kidney (HEK)) are as high as those obtained conventionally (Figure 3c; Figure S5b,c, Supporting Information); moreover, >90% cells identified as viable adherent ones in original images are isolated successfully in polygons, and >95% picked colonies are successfully transferred to (and expanded in) wells in conventional plates. These results show the Poisson limit can be “beaten”, with clones picked after 8 days in this case (instead of ≥2 weeks) due to the excellent optical clarity afforded by fluid walls. This approach also gives users confidence that a polygon contains only one cell/colony, so they can forego a second cloning round. More generally, any cell of interested in a complex population can be isolated (e.g., one with a particular morphology or expressing a fluorescent reporter).

### 2.5 Subculturing Cells Using Contactless Jetting

**Figure 4:**
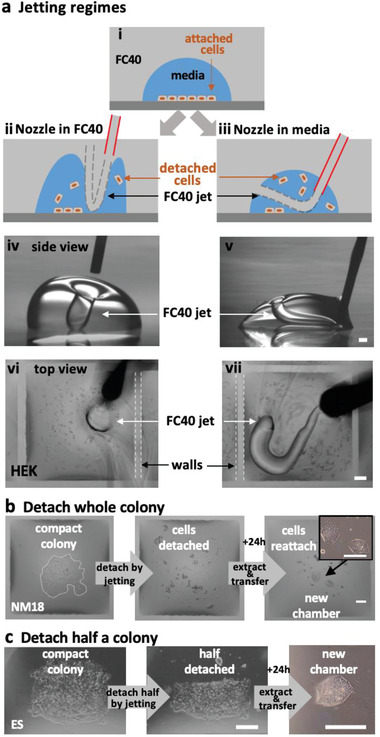
Dislodging attached living cells by jetting. Scale bars: 200 µm. a) Two jetting regimes (illustrated using a drop of media and a hand‐held jet viewed through a microscope; white dashes outline wall edges). i–iii) With the nozzle in FC40 or in media, a jet can depress the FC40:media interface so it plays on, and detaches, cells. iv,v). Side views of the two regimes operating on drops (volumes 3 and 1 µL) with circular footprints (without cells). FC40 jets are invisible. vi,vii) HEKs are plated in a 16 × 16 grid (as Figure 1b), grown for 24 h, and detached using the two regimes and a hand‐held nozzle. Remarkably, fluid walls survive. b,c) Detaching a whole or half a colony. A colony of NM18 (outlined by dotted white line) or ES cells growing in a chamber (as Figure 1b) are detached using a nozzle in FC40, detached cells removed and replated in a new chamber, where they grow.

The momentum of an FC40 jet can be insufficient to form a wall but sufficient to dislodge adherent cells from a dish without using trypsin or EDTA (think of a jet‐hose cleaning the bottom of a pool). There are two regimes. In one, the nozzle is placed in FC40 above media so jet momentum depresses part of the FC40–media interface to force it down so it plays on attached cells and detaches them (**Figure** **4**ai). The jet minimizes its area of contact with the aqueous phase when rebounding back to rejoin the overlay (Movies S6 and S7, Supporting Information). Whole colonies can be dislodged, extracted, and replated (Figure 4b). Half a colony can be retrieved, as the other half is kept as back‐up (Figure 4c; Movie S8, Supporting Information). Importantly, this allows a colony to be sampled several times as it grows to allow developmental and differentiation markers to be checked, without introducing the growth setbacks of completely removing and then replating the cells. Naturally, different cell types require different amounts of shear for detachment, with consequential effects on viability. For example, loosely attached HEKs and NM18 are easily detached as >95% remain viable, whereas tightly attached ES cells are more difficult to dislodge and ≈5% reattach and regrow (Figure S6a, Supporting Information). The jet also produces vortices in chambers that can be used to mix small volumes (e.g., 200 nL human blood; Movie S9, Supporting Information).

The second regime applies if the hydrophilic nozzle is lowered into media so it is wetted around its circumference. Now, jetted FC40 forms a squirming “worm” surrounded on all sides by media as it makes its way back to the overlay, usually through a “hole” in the chamber ceiling (Figure 4a, right; Movie S10, Supporting Information). Both regimes allow adherent cells to be subcultured without adding enzymes or chemicals, and mix microvolumes efficiently.

### 2.6 A Complex Perfusion Circuit with Constant Flow

**Figure 5:**
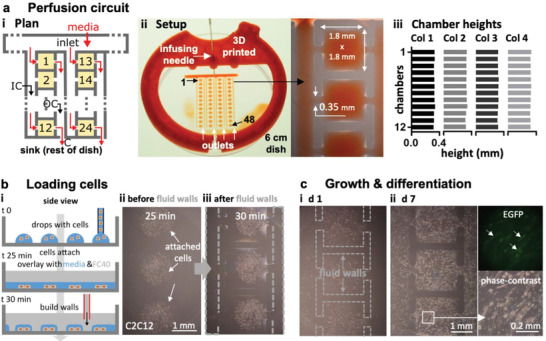
Perfusion circuit for continuous feeding 48 sets of differentiating myoblasts. a) Plan of the first two columns in the four‐column circuit i). Media (±dye) driven by an external pump into the inlet flows (red arrows) through an input conduit (IC), one cell chamber (chambers 1, 2, …, 24 are orange), an output conduit (OC), a choke (C), and out into the dish (the sink). Gray lines indicate FC40 walls. ii) Setup. Red dye is pumped into the inlet through a 3D‐printed adapter (red) that fits on a 6 cm dish, through 48 chambers, and out into the dish (zoom shows some chambers). iii) All chambers have similar maximum heights (average ± SD = 325 ± 6.3 µm; assessed using fluorescent dyes as in Figure S7 in the Supporting Information) showing that all experience similar pressures, shear stresses, and flows. b) Growth and differentiation of mouse C2C12 myoblasts. i) Workflow. Myoblasts are deposited in drops, the dish filled with media and FC40 added, and finally the circuit is jetted around them. ii,iii) Cells before/after building fluid walls. Dashed lines: edges of some walls. c) Images of chambers as media replenished (1 µL per day per chamber); myoblasts grow to fill chambers, form syncytia, and express EGFP‐DOK7 (day 7). Fluorescence and phase‐contrast zooms: arrows mark fluorescing syncytia each containing >20 nuclei with length >200 µm—indicative of differentiation into myotubes. The speed and extent of growth and differentiation is like that of cells grown conventionally.

We now illustrate the design, fabrication, and operation of a complex circuit that provides steady flows of fresh media for 7 days as cell grow in an array of chambers. Many conventional circuits doing this have been made,^[^ ^22^, ^23^, ^24^ ^]^ but they are custom‐built and difficult to integrate into workflows in biolabs. We designed one where every chamber receives fresh media. An external pump drives media through an input conduit (IC), on to one cell chamber (in a 4 × 12 array), and thence to an output conduit (OC), a choke (C), and finally into the sink (the rest of the dish). The choke acts to minimize pressure differences between input and sink, so equalizing flows through each cell chamber; **Figure** **5**ai). Heights in chambers were quantified by measuring the intensities given by a red dye or fluorescein; all cell chambers yielded similar intensities (Figure 5aii; Figure S7, Supporting Information). As fluid walls/ceilings morph during flow to reflect internal pressures, the demonstration that all chambers (which have identical footprints) have similar heights (and so similar volumes), confirms that all experience similar pressures, shear stresses, and flows (Figure 5aiii; Figure S7, Supporting Information). This illustrates another advantage of fluid walls: chamber/conduit height serves as an inbuilt pressure and flow indicator.

Circuit operation is demonstrated using mouse C2C12 myoblasts that can differentiate over 7 days into mature myotubes; they fuse to form syncytia and express components of the neuromuscular junction at the expected rate—including the adaptor protein, DOK7—a differentiation marker which in this case is tagged with EGFP.^[^ ^25^ ^]^ The undifferentiated (nonfluorescent) myoblasts can be added to each chamber immediately after fabrication; then, as chambers have Laplace pressures lower than flanking conduits, deposited cells remain in chambers. While this method is quick, it has the drawback that cells are exposed to equilibrating flows (and shear stresses) as they settle, and this can adversely affect some cell types (not shown). Therefore, cells are deposited as drops (in air) on a virgin dish at positions corresponding to those where the 48 chambers will eventually be built; on incubation, cells attach under shear‐free conditions (Figure 5bi). Next, media is added to the whole dish to merge drops, FC40 overlaid, and the circuit built around the 48 groups of living cells (Figure 5bii,iii). The dish is now placed in a CO_2_ incubator, and fresh media slowly pumped at 0.55 nL s^−1^ into the circuit over 7 days; then, media in each chamber is exchanged at a rate of 1 µL per day. Subsequently, cells grow and differentiate into myotubes, form syncytia, and become fluorescent (Figure 5c; Figure S7d, Supporting Information). This shows that flows through a complex circuit can be equalized by judicious design, and that cells flourish and differentiate at the same rate and to the same extent as those grown conventionally. Although growth increases numbers and some cells migrate toward inlet and outlet channels to eventually meet those from neighboring chambers, microscopic analysis is easily restricted to those cells that remain in any particular chamber. If required, any chamber can be disconnected from the rest of the circuit by building new FC40 walls across input and output conduits, and its contents extracted for downstream analysis.

## 3 Conclusion

We describe a general and rapid method to fabricate microfluidic circuits on a standard polystyrene Petri dish. A microjet of FC40 is projected through FC40 and media on to the bottom of a dish; as FC40 hits the dish, it washes away media to leave a liquid wall of FC40 tightly “pinned” to the substrate by interfacial forces (Figure 1a). Circuits with almost any imaginable 2D shape can be built in seconds to minutes (Figure 1b–h). We establish conditions required to build stable fluid walls (Figure 2), drive flow through conduits without using external pumps (Figure S3, Supporting Information), and create “valves” that can be opened and closed at will (Figure S4, Supporting Information). We develop an approach for cloning dilute suspensions of mammalian cells that can beat the Poisson limit in the sense that almost every cell in the initial population is confined within its own fluid wall (Figure 3). We extend the approach to subculture attached cells without adding trypsin or EDTA (Figure 4), and to feed arrays of mammalian cells over long periods (Figure 5). Unfortunately, evaporation from static volumes of less than ≈1 nL inevitably limits construction of ever‐smaller devices (even though the solubility of water in FC40 is <7 ppm by weight).

## 4 Experimental Section

##### General Reagents

All reagents and materials were purchased from Sigma Aldrich (St. Louis, Missouri), unless otherwise stated. FC40^STAR^ (iotaSciences Ltd, Oxfordshire, UK) is FC40 treated using a proprietary method that improves wall formation by jetting, and throughout the term “FC40” is used to refer to it. Water‐soluble dyes (e.g., Allura Red, toluidine blue, resazurin) are used where indicated.

##### Cells and Cell Culture

Whole human blood (anticoagulated with EDTA) was obtained from anonymous blood donors through the National Health Service (NHS) Blood and Transplant Service (Oxford, UK); ethical approval was provided by The Interdivisional Research Ethics Committee of Oxford University (R63966/RE001).

Adherent human embryonic kidney cells (HEKs) were grown in DMEM (Gibco, Gaithersburg, MD) + 10% FBS; these HEKs were genetically modified reporter cells (NF‐kB/293/GFP‐Luc Transcriptional Reporter Cell Line; System Biosciences, catalogue number TR860A‐I), but reporter activity is not relevant here.

Mouse embryonic stem (ES) cells (EK.CCE line, derived from a single XY blastocyst‐stage embryo of strain 129/Sv/Ec)^[^ ^26^ ^]^ were routinely cultured in DMEM supplemented with 15% FBS, 1% penicillin/streptomycin (Gibco, #15140122), 0.1 × 10^−3^ m 2‐mercaptoethanol, 1% glutamine (Invitrogen, Loughborough, UK), 1% minimum essential media nonessential amino acids (Gibco), 1 × 10^−3^ m sodium pyruvate, and 1000 U mL^−1^ leukemia inhibitory factor (LIF, ESGRO; Merck, Darmstadt, Germany; #L5158) on gelatin‐coated plates (Merck, #ES‐006‐B). Such plates were used for Figure 3c and Figure S5b (Supporting Information).

Mouse mammary tumor cells (NM18, a derivative of the NMuMG line)^[^ ^27^ ^]^ were cultured in DMEM supplemented with 10% FBS, 1% penicillin/streptomycin, and 0.1% insulin (Sigma Aldrich, #10516).

C2C12, an immortalized mouse myoblast,^[^ ^25^ ^]^ were cultured in DMEM + 15% FBS. For the perfusion circuit in Figure 5, cells were seeded in DMEM + 15% FBS, and the FBS was reduced to 6% after 4 days, and to 2% after 5 days.

##### Printing and Operating Circuits

All circuits were made on tissue‐culture‐treated surfaces—60 or 35 mm circular dishes (Corning, Merck product 430166 or #430165) or rectangular uniwell plates with overall dimensions of 96‐well plates (Thermo Fisher Scientific, Waltham, MA) using custom‐written software (see also below) and modified “Freestyle” or “Pro” printers (iotaSciences Ltd, Oxfordshire, UK). Each printer consists of a three‐axis traverse, which controls movement of stainless‐steel needles (Adhesive Dispensing Ltd, Milton Keynes, UK)—usually a 60 µm inner‐diameter nozzle jetting FC40 (outer diameter 0.5 mm), and a 0.5 mm outer‐diameter needle (inner diameter 0.25 mm) used to add/remove media to/from chambers. The two needles are each connected via Teflon tubing to a programmable syringe pump. The Freestyle accepts 60 and 35 mm dishes, while the Pro has a larger working area and less mechanical backlash, so it was used to print the circuits in Figure 1d,e,g, Figures S1 (Supporting Information), and Figure 5a. Flows through circuits were driven by programmable syringe pumps (Harvard PhD Ultra I/W) connected via Teflon tubing to blunt stainless‐steel dispensing needles (outer diameter 0.5 mm); needles were lowered through FC40 into the circuit at the appropriate position, when fluid walls spontaneously self‐seal around inserted needles. It is anticipated that fluidic arrangements with even finer features can be jetted using three‐way traverses with greater accuracy and precision.

DMEM plus 10% FBS was used to make all grids and circuits; when the term “medium” is used, it should be assumed that 10% FBS is also present. Routinely, to prepare a grid or circuit, 1 mL medium is pipetted into a 60 mm dish to wet the entire bottom surface and ≈0.9 mL retrieved to leave a thin film. This is then overlaid with 2–3 mL FC40 and the dish placed on the printer. Standard conditions used for jetting FC40 when making most circuits were *D*_nozzle_ = 60 µm, *H* = 0.4 mm, $\dot{Q}$ = 8 µL s^−1^, and *V*_traverse_ = 10 mm s^−1^. A tightly fitting 3D‐printed circular sleeve around each dish acts as a positioning ring to ensure the dish can be removed and added back on to the printer in the original orientation. The adapter in Figure 5aii used to hold the needle feeding the perfusion circuit in a 60 mm dish was 3D printed with polylactic acid (rigid.ink, Wetherby, UK).

Patterns in Figure 1c,d,e,g,h, Figure S1 (Supporting Information), and Figure 5a were constructed after reproducing the desired circuit in Inkscape (inkscape.org) and conversion to G‐code, the programming language used by the printers. Patterns for Figure 1d,e,g,h were obtained from Wikipedia, CanStockPhoto, http://www.udel.edu and WikiArt, respectively.

For Figure 3 and Figure S5 (Supporting Information), ImageJ^[^ ^28^ ^]^ was used to detect and highlight attached single cells. Images were then processed in MATLAB (MathWorks, Cambridge, UK), where coordinates of single cells were recorded and Voronoi diagrams subsequently generated. The code used is available in Supplementary Materials. Finally, Inkscape was used to convert the diagrams into the G‐code used to print polygons. Areas and centroids of each polygon were also computed, and the volumes to be added to each polygon calculated using the formula *V*_cap_ = (*A*_wetted_ /3.7)^3/2^, where *V*_cap_ is the maximum volume that can be added to a polygon assuming an advancing contact angle of 50°^[^ ^6^ ^]^ and *A*_wetted_ is the area of the polygon. The same volumes were removed from, and added to, each polygon when refreshing media, and during colony retrieval (which involved emptying chambers, PBS addition and removal, trypsin addition, and removal of the resulting cell suspension). Retrieved cells were plated in 12‐well plates (Greiner Bio‐One, Kremsmünster, Austria; #665180) and allowed to reattach and grow.

##### Imaging

Images of dishes in Figure 1b,d,e,g,h, Figures S1 and S2c (Supporting Information), Figure 3a, Figure S3c (Supporting Information), Figure 4a, Figure S4c, Figure 5a, and Figure S7a (Supporting Information) were taken using a digital SLR camera (Nikon D610). All insets in these figures, and fluorescence images in Figure 5c and Figure S7d (Supporting Information) were collected using a zoom lens and digital SLR camera (Nikon D7100 DSLR) connected to an epi‐fluorescent microscope (Olympus IX53; 1.25×, 4×, 10×, and 25× objectives) with translation stage and overhead illuminator (Olympus IX3 with filters).

For Figure 3, 35 mm dishes were imaged using a phase‐contrast live‐cell imaging system and a 4× objective (IncuCyte Zoom, Sartorius, Gottingen, Germany).

For fluorescent images of cells in Figure 5c and Figure S7d (Supporting Information) an exposure time of 1 s was used. Quantification of EGFP intensity was done in ImageJ by subtracting the background and averaging the intensity across the entire image. Brightness was then increased by 40% for these images for a better visualization of cells expressing EGFP.

Movies S3, S6, S7, and S10 (Supporting Information) were taken using a pendant‐drop instrument (First Ten Angstroms 1000, Cambridge, UK).

##### Statistical Analysis

Statistical analysis was performed using GraphPad Prism (San Diego, CA).

For Figure 2b each data point represents an average of *n* = 9 tests performed across three different dishes.

For Figures 3c and 5 dishes were used for each cell line and each of the two conditions (with and without fluid walls). To assess whether there is a significant difference between cloning efficiencies for the two conditions, unpaired *t* tests were performed.

For Figure S7bii (Supporting Information), a second order polynomial was fitted to the experimental data. This polynomial was then used to infer heights of chambers from fluorescence intensity.

## Conflict of Interest

Oxford University Innovation—the technology transfer company of The University of Oxford—has filed provisional patent applications on behalf of C.S., P.R.C., and E.J.W. partly based on this study. P.R.C. and E.J.W. each hold equity in, and receive fees from, iotaSciences Ltd, a company exploiting this technology; iotaSciences Ltd also provided printers, FC40^STAR^^®^, and scholarships for C.S. and C.D.

## Author Contributions

C.S., P.R.C., and E.J.W. designed research; C.S., N. S‐K., C.D., and E.J.W. performed experiments; and all authors wrote the paper.

## Supporting information

Supporting Information

Supplemental Movie 1

Supplemental Movie 2

Supplemental Movie 3

Supplemental Movie 4

Supplemental Movie 5

Supplemental Movie 6

Supplemental Movie 7

Supplemental Movie 8

Supplemental Movie 9

Supplemental Movie 10
