# Supplementary material for: Jet‐Printing Microfluidic Devices on Demand
Source: Adv Sci (Weinh). 2020 Oct 26;7(23):2001854. doi: 10.1002/advs.202001854 (PMC7709972; doi:10.1002/advs.202001854)
Supplement: Supplementary file 1 — Supporting Information [file ADVS-7-2001854-s001.pdf]

WILEY-VCH

© 2020 Wiley-VCH GmbH

ADVANCED SCIENCE
Open Access

Supporting Information

for *Adv. Sci.,* DOI: 10.1002/advs.202001854

Jet-Printing Microfluidic Devices on Demand

Cristian Soitu, Nicholas Stovall-Kurtz, Cyril Deroy, Alfonso A. Castrejón-Pita, Peter R. Cook, and Edmond J. Walsh

© 2020 Wiley-VCH GmbH

Supporting Information

Title Jet-printing microfluidic devices on demand

Author(s), and Corresponding Author(s)*: Cristian Soitu, Nicholas Stovall-Kurtz, Cyril Deroy, Alfonso A. Castrejón-Pita, Peter R. Cook*, and Edmond J. Walsh*

Supplementary figures

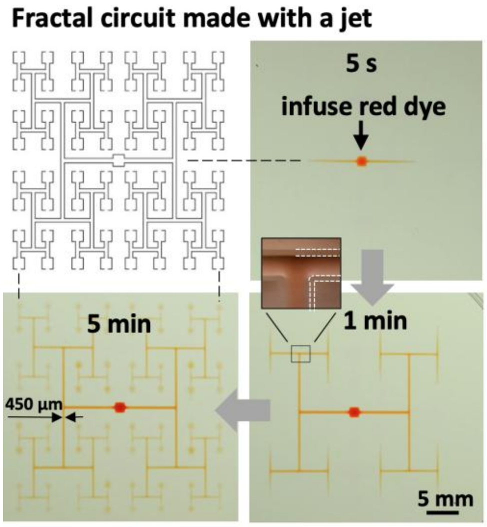

Figure S1. Making a fractal circuit using a jet. The jet provides great accuracy and precision, as the jetting needle is held by the traverse at a defined *x*,*y*,*z* position (so there is no play in the system), and it contacts neither media nor dish. This circuit is constructed with high accuracy in ~6 min from T-shaped segments (plan shown at top left; conduits have footprint widths of 450 µm) connecting a central input to 64 outlets and the rest of the dish (the sink). Three images show the circuit in operation; red dye was infused into the input and it reaches each outlet simultaneously, showing that all branches have similar dimensions. Inset: one junction with some wall edges highlighted white.

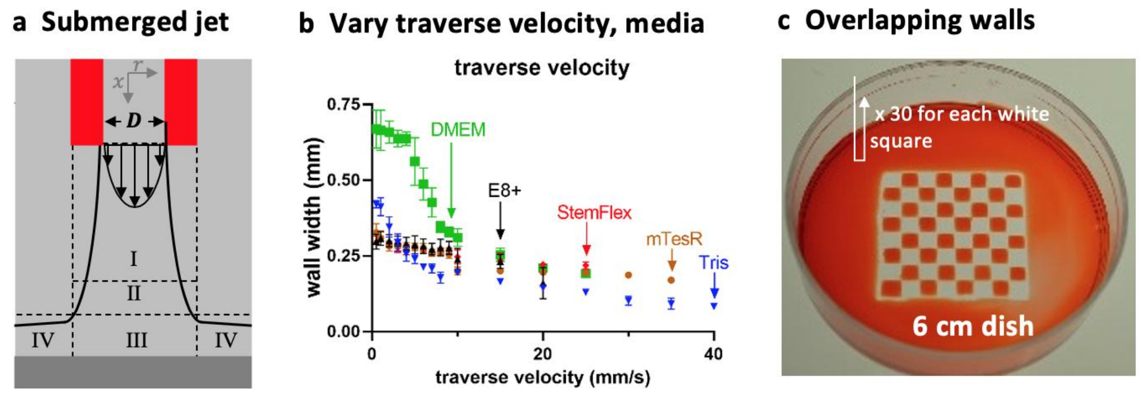

Figure S2.

a. Flow profiles in/around a submerged jet impinging on a flat substrate (Deshpande and Vaishnav, 1982). Five arrows illustrate the flow profile on emergence from the nozzle, and then there are 4 regions of jet flow assuming a fully-developed exit-flow velocity-profile. I: free jet region where jet diameter progressively increases. II: transition-to-wall zone. III: stagnation zone. IV: wall-jet region. In our case, the substrate is initially covered with media and removed by jet momentum and the shearing force in the wall-jet zone as the jet traverses.

b. Effect of traverse velocity on wall width using different media (H = 0.5 mm, $\dot{Q}$ = 8 µl/s; 4 mg/ml resazurin dye added to 20 mM TrisHCl). No media tested gave stable walls with $V_{traverse}$ >35 mm/s (as dwell time over any one area in the dish is too short for the jet to remove media from the surface).

c. Building wide overlapping walls (3 x 3 mm) by jetting through media + dye.

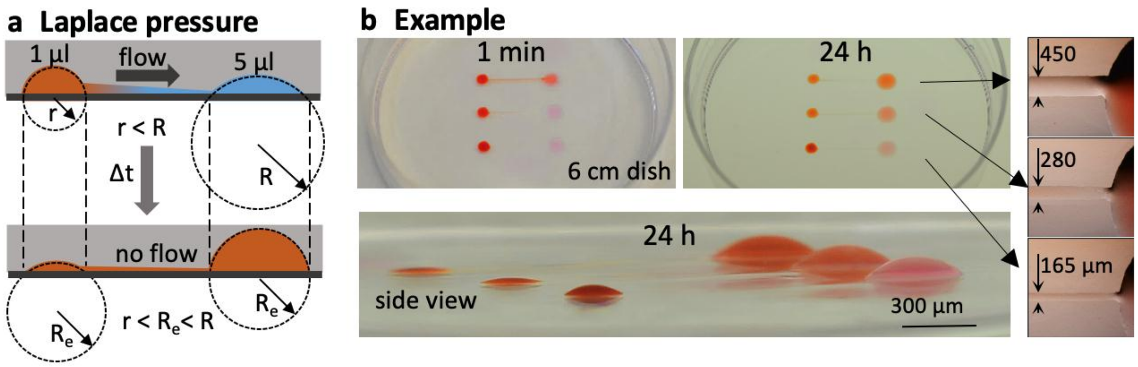

Figure S3. Controlling flow through conduits with different widths using differences in Laplace pressure.

a. Laplace pressure – principle. Top: a difference in Laplace pressure spontaneously drives flow as drops have different radii of curvature (r<R). Bottom; flow ceases when both drops have the same curvature and radius at equilibrium, R_e.

b. Example. Each of the 3 circuits was made by jetting one continuous wall; all 3 are initially identical (left- and right-hand chambers have diameters of 2.5 and 5 mm respectively) except for conduit width which varies from 450-165 µm. Red dye (5 µl) is added to left-hand drops to increase Laplace pressure to drive flow to the right. After 1 min, some dye in the upper circuit (but not others) reaches the right-hand drop. After 24 h, the left-hand upper drop is nearly flat (and so empty) as essentially all dye has been discharged through the widest conduit to the right-hand one. In contrast, the left-hand lower drop remains rounded and continues to drive flow through the narrowest conduit. Insets: zooms of conduits with widths indicated.

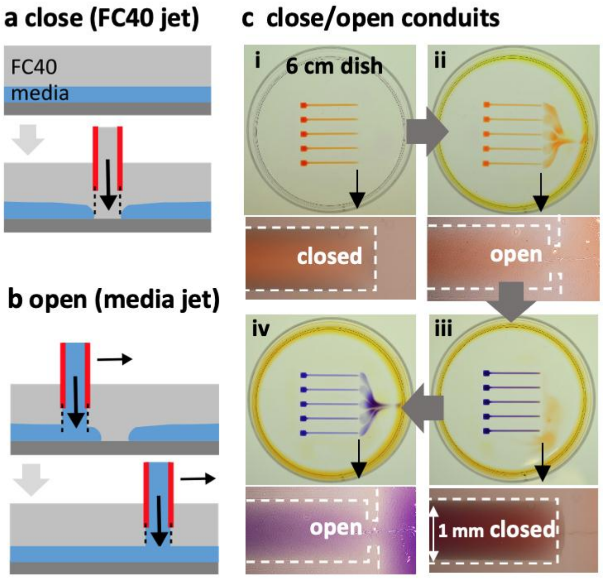

Figure S4. A microfluidic valve.
a. Closing an open conduit. A new wall across a conduit is built by jetting FC40 (nozzle moves into page).
b. Opening a closed conduit. A wall across a conduit is removed by jetting media through the wall (nozzle moves to right).
c. Example. Insets show conduit ends, with dashed lines marking FC40 walls. (**i**). Five continuous FC40 walls were printed to create 5 identical circuits (each a reservoir with a 2 x 2 mm footprint connected to a conduit 1 mm wide), and 2 µl red dye added to each reservoir. Red dye is confined to each circuit as the surrounding FC40 wall is continuous. (**ii**) Media was jetted (H = 0.4 mm, $\dot{Q}$ = 8 µl/s, $V_{traverse}$ = 15 mm/s) to open end walls. Red dye spills out into the dish (which acts as a sink, as it has a low Laplace pressure). (**iii**) Ends of conduits were closed by jetting FC40 (conditions as for media) to rebuild walls, and 2 µl blue dye added to reservoirs to test for leaks. No blue dye escapes, showing new walls provide perfect seals. (**iv**) Reopening end walls allows escape of blue dye. The pattern indicates flow through each end is similar, so openings have equal widths.

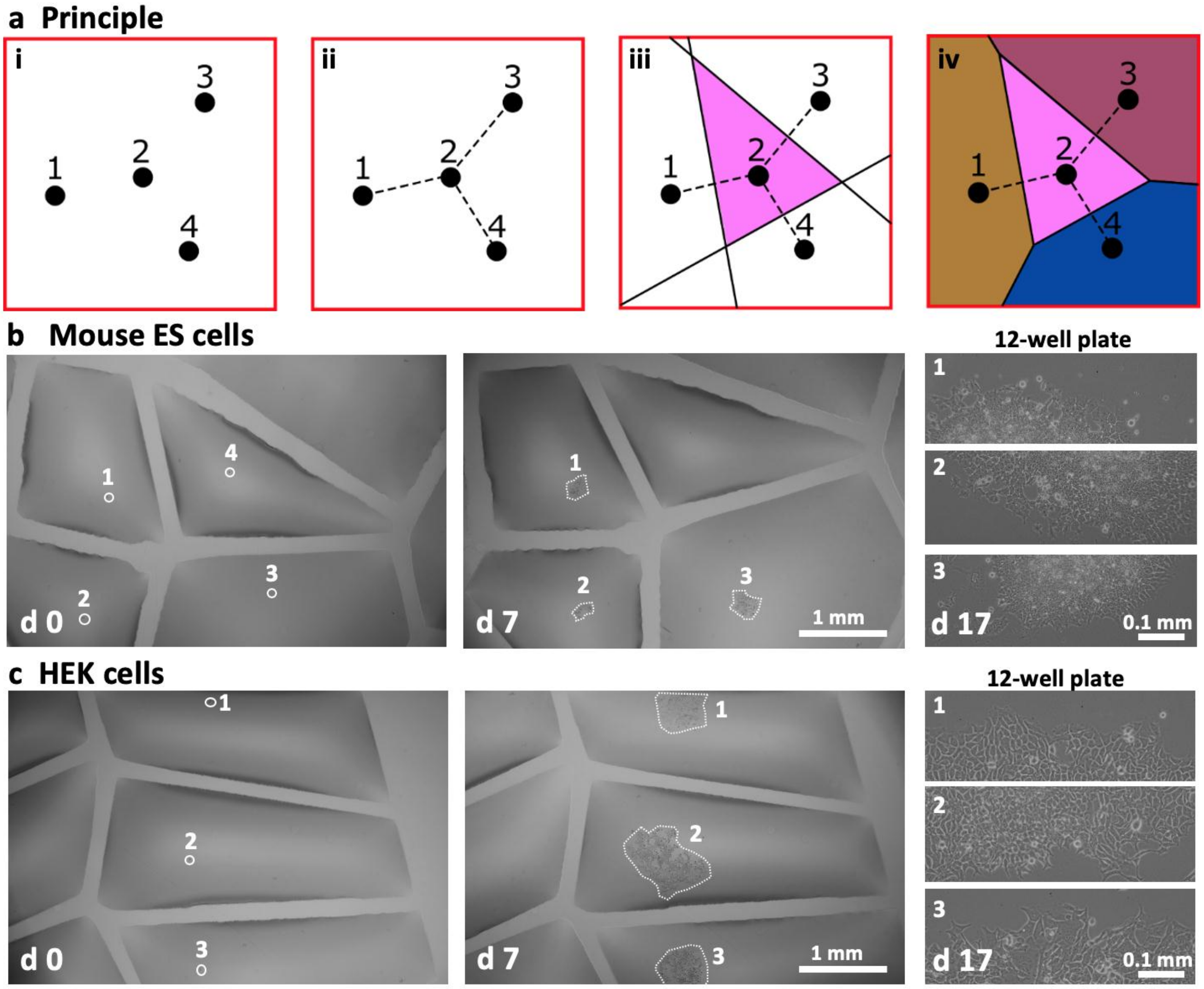

Figure S5. Drawing Voronoi diagrams, and examples of their use.
a. Principle. (i) In this example (4 points randomly-distributed in a square), (ii) we draw straight lines between each point (here shown for point 2) and its nearest neighbors. (iii) The corresponding Voronoi polygon is formed using half-plane intersections for all lines. (iv) The same procedure is applied for other points to produce the Voronoi diagram.
b, c. Example Voronoi diagrams used during cloning of ES and HEK cells. Details are as Figure 3, and each pair of images on the left show the same region of the dish immediately after plating cells (d 0; circles surround single cells), and prior to picking colonies (d 7; dotted lines outline colonies). Right-hand panels show views of picked colonies growing in conventional 12-well plates at d 17. Note that, in principle, the local concentration of autocrine factors secreted by cells might be different in polygons with different volumes, and as such a concentration might be many-fold higher than that in a well in a 96-well plate (as volumes are about 1,000 times smaller), and as a higher concentration of autocrine factors might increase cloning efficiency, one might expect improved efficiencies in Voronoi polygons compared to well plates. However, in practice, cloning efficiencies of 3 cell types tested in polygons and conventional plates proved to be similar (Fig. 3c).

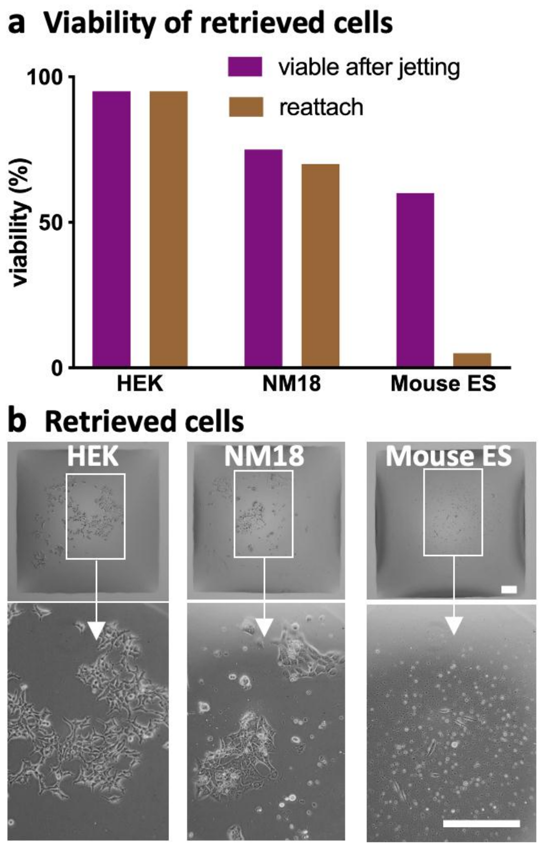

Figure S6. Cell viability after detachment by FC40 jetting. Cells (HEK, NM18, ES) in single colonies in a chamber (as in Figure 1b) were dislodged by FC40 jetting with the nozzle in FC40 and detached cells retrieved from the chamber; after assessing cell viability by trypan-blue staining, cells were transferred to new chambers and the percentage reattaching assessed.
a. Viability of retrieved cells. As might be expected, viability and reattachment rates depend on jet momentum required for detachment. Thus, loosely-attached HEKs are easily detached by jetting to remain viable and reattach, whereas tightly-attached ES are detached with difficulty and have poorer viabilities and reattachment rates**.**
b. Images of retrieved cells 24 h after replating in new chambers. Bars: 200 µm. Zooms show magnifications.

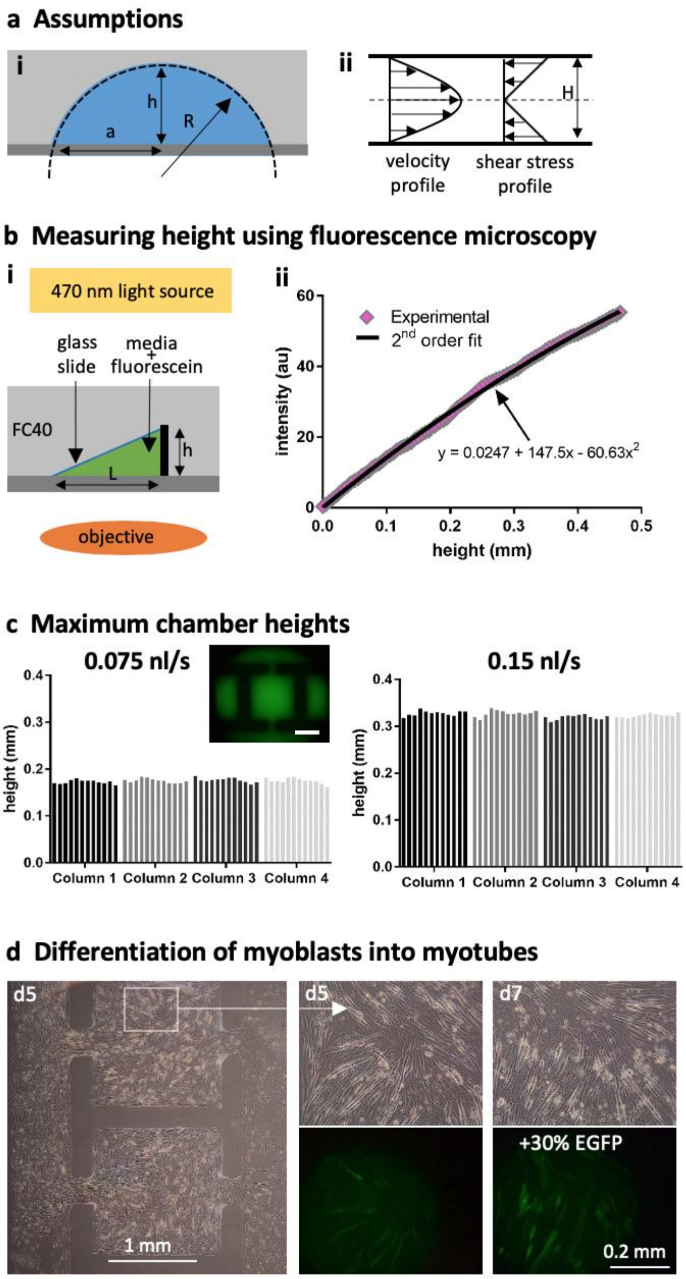

Figure S7. Confirming that cells in different chambers in the perfusion circuit in Figure 5 experience the same pressures and velocity profiles (and so rates of media replenishment and shear stresses).

a. Assumptions. (**i**) The 3D geometry of a cell chamber is complex, resembling that of a spherical cap sitting on a rectangular base;[7] for simplicity, we assume it is a perfect cap of a sphere (2a = chamber width, R = sphere radius). (**ii**) Then, the velocity and shear-stress profiles between bottom and top of any section through a chamber are functions of height, H, at that point in the section. Identical heights in all chambers indicate that cells in each chamber experience similar pressures, shear stresses, and flows.

b. Measuring maximum chamber height using fluorescence microscopy. (**i**) Construction of a calibration curve relating fluorescence intensity to height of fluorescein. A mixture of cell media and fluorescein is added between a glass slide propped against a pillar (height, h) and the bottom of a Petri dish, FC40 overlaid, the construct imaged, and the intensity at each point along the slide recorded. (**ii**). Relation between fluorescence intensity (arbitrary units, au) and height (calculated knowing dimensions of the triangle in (i)). Black line: best fit of a second-order polynomial.

c. Maximum chamber heights in circuits perfused at different rates. Media plus fluorescein was perfused through chambers in circuits ($\dot{Q}$ = 0.075 and 0.15 nl/s; data for 0.15 nl/s is reproduced from Figure 5aiii), fluorescence images collected (inset: fluorescence image of part of a circuit, bar = 1 mm), and chamber heights calculated using the calibration curve. Average heights were 175 µm ± 5 µm and 325 µm ± 6.3 µm SD at 0.075 and 0.15 nl/s, respectively. This indicates that for a given circuit and flow rate, heights of different chambers are similar, and that chamber height changes with flow rate (as fluid walls morph above unchanging footprints to accommodate the pressure difference). As chamber heights reflect both $\dot{Q}$ and velocity profiles, each chamber in a circuit experiences the same rate of media replenishment and shear stress.
d**.** Control test to show the increase of EGFP signal with differentiation. C2C12 cells were cultured for 7 d in the perfusion circuit. Insets show phase-contrast and fluorescence images of the same location inside a chamber at d 5 and d 7. For an exposure time of 1 s, the average intensity of signal per pixel increases by 30% between the two time points, indicating increased expression of the tagged DOK7 (and so differentiation into a myotube).

Supplementary Movies

Movie 1. Jetting a grid. The movie runs at 4x speed. A Petri dish (6 cm) – filled with a skim of media mixed with blue dye plus an FC40 overlay – sits on a printer, and the nozzle of a hollow needle (D_{nozzle} = 60 µm) is held by a 3-way traverse in FC40 above media in the dish (H= 0.4 mm). The movie begins as the pump infuses FC40 ($\dot{Q}$ = 8 µl/s) and the traverse traces the path of a grid ($V_{traverse}$ = 10 mm/s). The invisible jet of FC40 pierces the layer of media, pushing it aside to contact the substrate. As FC40 wets polystyrene preferentially, it remains firmly pinned to it. A grid with 256 aqueous chambers, each surrounded by transparent FC40 walls, is created in <2 min.

Movie 2. A 16 x 16 grid withstands violent agitation. The movie runs in real time. A grid like the one in Figure 1b was constructed, each chamber filled with 600 nl red dye, and the dish placed on a shaker.

Movie 3. Jetting Novec 649 from a nozzle submerged in FC40 confirms that jet width increases with distance from the nozzle. The movie runs in real time (10 frames/sec). This side view depicts a nozzle of a 34G needle (D_{nozzle} = 80 µm, outer diameter 220 µm) submerged in FC40. Then, another fluorocarbon (i.e., Novec 649) – chosen because it is miscible with FC40 but has a different refractive index so interfaces between the two transparent liquids can be seen – is jetted from the nozzle (D_{nozzle} = 60 µm) through FC40. Jet width increases with distance from the nozzle (see also Deshpande and Vaishnav, 1982). Turbulence at the end of the movie results from Novec 649 rebounding from the substrate.

Movie 4. A microfluidic valve. The movie plays at 1x or 4x real time as indicated. It illustrates the experiment shown in Figure S4. The 6 cm dish initially contains 5 identical circuits, each a reservoir connected to a conduit 1 mm wide with a dead-end, and each bounded by one continuous wall. Then, the printer adds 2 µl red dye to each reservoir (walls are intact and dye stays within each circuit), jets media to open end walls (red dye spills out into the dish), jets FC40 to rebuild end walls, adds 2 µl blue dye to reservoirs to test for leaks (no blue dye escapes), and jets media to reopen end walls (so now blue dye does escape).

Movie 5. Visualizing a Voronoi diagram. The movie is speeded up 4x, and illustrates the kind of experiment shown in Figure 3a where an area of 20 x 20 mm in a 35 mm dish was subdivided into 64 Voronoi polygons, each containing one dot. Polygons are initially invisible due to the low volume each contains. The movie shows addition of media + black dye by a printer through FC40 to each polygon (added volume is proportional to polygon area, and ranges from 0.25–2.8 µl).

Movie 6. FC40 from a nozzle held above drops of media deforms FC40–media interfaces so a depressed section plays on the bottom. The movie runs in real time (10 frames/sec). It depicts a side view of a nozzle (a 34G needle, D_{nozzle} = 80 µm, outer diameter 220 µm) held below the surface of FC40 but above drops of media (shaped like caps of spheres with volumes of 1–3 µl) sitting in a dish. The movie begins as the nozzle jets FC40, and traverses above drops. Although the jet is invisible, it deforms sections of the FC40–media interface in each drop as it passes. These sections are forced down to the substrate, and can be used to detach adherent cells.

Movie 7. Jetting Novec 649 from a nozzle held above a drop of media deforms the FC40–media interface so a depressed section plays on the bottom. The movie runs in real time (10 frames/sec). In Movie S3, FC40 is invisible, and there is no indication of any turbulent flow

after the jet leaves the drop. Here, the jet contains another fluorocarbon (Novec 649) that is miscible with FC40 but has a different refractive index so interfaces between all 3 liquids can be seen. Initially the nozzle (34G needle, D_{nozzle} = 80 µm, outer diameter 220 µm) is held below the surface of FC40 but above a 1 µl drop of media. Novec 649 is then jetted on to the drop, the nozzle traverses back and forth above the drop, and a depressed section of the FC40–media interface plays on the bottom.

Movie 8. Detaching half a stem-cell colony growing in a chamber in a grid. The movie runs in real time, and images were collected on a microscope from above. The movie depicts removing part of a living colony of mouse ES cells from a chamber (as in Figure 1b) using a hand-held FC40 jet (D_{nozzle} = 60 µm; $\dot{Q}$ = 9 µl/s). The nozzle is positioned in FC40 and above the media–FC40 interface throughout, and the jet depresses a section of the interface so it plays on the bottom of the chamber to detach some cells in the colony. Figure 4c illustrates accurate removal of half a colony using the printer, but then imaging through a microscope from above (as in this movie) is impossible.

Movie 9. Mixing blood using the regime described in Figure 4a where the nozzle is held below an FC40:aqueous interface. The movie runs in real time. A grid like the one in Figure 1b was made, 0.2 µl whole human blood added to two chambers, and a manually-held jet used to create vortices in chambers, and so mix the blood.

Movie 10. Jetting FC40 from a hydrophilic nozzle submerged in a drop of media yields a ‘squirming worm’ (which can be used to detach cells). The movie runs at 5x speed (10 frames/s). This side view depicts a 1 µl drop of media (initially shaped like the cap of a sphere) under FC40, and the hydrophilic nozzle (D_{nozzle} = 80 µm) is below the surface of FC40 but above the drop. After 1s, the pump jets FC40 ($\dot{Q}$ = 10 µl/s) on to the drop to deform the FC40–media interface. After 5 s, the hydrophilic nozzle is lowered towards the drop. By 12 s, the nozzle enters the drop and is wetted around its circumference; consequently, the FC40 jet is surrounded on all sides by media as it leaves the drop to rejoin the overlay (which it does at the back, and so out of sight). After ~24 s, flow rate is increased ($\dot{Q}$ = 15 µl/s), and the jet rearranges and extends from the nozzle to the right to appear as a ‘squirming worm’. By 35 s, the ‘exit hole’ of the jet from the drop is clearly visible. At ~85 s, the flow rate is reduced ($\dot{Q}$ = 10 µl/s), and at ~125 s increased again ($\dot{Q}$ = 15 µl/s). The worm continues to squirm in the drop until the end of the movie.

Code used to create Voronoi diagrams

```
%read image with single cells to be analysed.
image = imread('single_cells.tif');

%get the size of the image
[rows, columns, numberOfColorChannels] = size(image);

%show the image
imshow(secondlevelTIFF);

%project a grid on top of the image to help with the identification of single cells
grid_lines_every = 1000;
for row = 1 : grid_lines_every : rows
  line([1, columns], [row, row], 'Color', 'r');
end
for col = 1 : grid_lines_every : columns
  line([col, col], [1, rows], 'Color', 'r');
end

%record position of each cell
X = []; Y = []; points = []; i = 0; datacursormode on;
while (i < 1000)
    dcm_obj = datacursormode(figure(1));
    pause
    cursor_info = getCursorInfo(dcm_obj); position = cursor_info.Position;
    X = [X, position(1)]; Y = [Y, rows - position(2)]; i = i + 1;
end
datacursormode off

%store positions in txt files
dlmwrite('Xpos.txt', X); dlmwrite('Ypos.txt', Y);

%apply Voronoi diagram to the set of points
voronoi(X,Y);
```
